# VWCE as a potential biomarker associated with immune infiltrates in breast cancer

**DOI:** 10.1186/s12935-021-01955-3

**Published:** 2021-05-21

**Authors:** Qin Huo, Zhenwei Li, Siqi Chen, Juan Wang, Jiaying Li, Ni Xie

**Affiliations:** 1 Biobank, Shenzhen Institute of Translational Medicine, Shenzhen Second People’s Hospital, First Affiliated Hospital of Shenzhen University , Shenzhen, 518035 China; 2 Department of Clinical Medicine , University of South China , Hengyang , 421001 China

**Keywords:** VWCE, Tumor-infiltrating, Biomarker, Breast cancer

## Abstract

**Purpose:**

Von Willebrand Factor C and EGF Domains (VWCE) is an important gene that regulates cell adhesion, migration, and interaction. However, the correlation between VWCE expression and immune infiltrating in breast cancer remain unclear. In this study, we investigated the correlation between VWCE expression and immune infiltration levels in breast cancer.

**Methods:**

The expression of VWCE was analyzed by the tumor immune estimation resource (TIMER) and DriverDB databases. Furthermore, genes co-expressed with VWCE and gene ontology (GO) enrichment analysis were investigated by the STRING and Enrichr web servers. Also, we performed the single nucleotide variation (SNV), copy number variation (CNV), and pathway activity analysis through GSCALite. Subsequently, the relationship between VWCE expression and tumor immunity was analyzed by TIMER and TISIDB databases, and further verified the results using Quantitative Real-Time PCR (RT-PCR), Western blotting, and immunohistochemistry.

**Results:**

The results showed that the expression of VWCE mRNA in breast cancer tissue was significantly lower than that in normal tissues. We found that the expression level of VWCE was associated with subtypes, estrogen receptor (ER), progesterone receptor (PR), and human epidermal growth factor receptor-2 (HER2) status of breast cancer patients, but there was no significant difference in the expression of VWCE was found in age and nodal status. Further analyses indicated that VWCE was correlated with the activation or inhibition of multiple oncogenic pathways. Additionally, VWCE expression was negatively correlated with the expression of STAT1 (Th1 marker, r = − 0.12, p = 6e−05), but positively correlated with the expression of MS4A4A (r = 0.28, p = 0). These results suggested that the expression of VWCE was correlated with immune infiltration levels of Th1 and M2 macrophage in breast cancer.

**Conclusions:**

In our study, VWCE expression was associated with a better prognosis and was immune infiltration in breast cancer. These findings demonstrate that VWCE is a potential prognostic biomarker and correlated with tumor immune cell infiltration, and maybe a promising therapeutic target in breast cancer.

**Supplementary Information:**

The online version contains supplementary material available at 10.1186/s12935-021-01955-3.

## Background

Breast cancer is the most common type of cancer and second death-leading cancer type in women [1–3]. Despite years of research, the 5-year survival rate of breast cancer patients is still very low [4]. As reported, the immune infiltration of tumor microenvironment is related to the survival rate of many solid tumor patients [5]. Recently, several reports have emphasized in tumor-infltrating lymphocytes (TILs) can improve the prognosis of various types of tumors, including colon [6], ovarian [7], and renal cell cancer [8]. TILs are composed of multiple lymphocytes, such as B cells, CD4^+^ T cells, CD8^+^ T cells, macrophages, NK cells, and dendritic cells [9]. The different effects of immune cells on tumors depends on the tumor environment [10]. Stanton S E et al. demonstrated that TILs played an important role in tumor-related immune response and improved clinical outcomes in breast cancer [11]. However, the clinical impact of immune cells in breast cancer remains poorly understood. In view of the high morbidity and mortality of breast cancer, it has become a current research trend to investigate molecular mechanisms to determine potential molecular biomarkers and provide evidence for early diagnosis, prevention, and personalized treatment.

Von Willebrand Factor C and EGF Domains (VWCE, also known as URG11) is located on the long arm of chromosome and is predicted to be a secreted protein-encoding a 99 kDa protein, it consists of five chordin like cysteine repeats and a C-type lectin domain [12]. As a newly identified gene, VWCE has been reported to be highly expressed in pancreatic cancer tissues. It can inhibit the expression of EMT markers and the invasion of pancreatic cancer cells by RNA interference [13]. Besides, it is reported that VWCE is closely related to tumor node metastasis (TNM) stage and lymph node metastasis. VWCE promotes the G1/S phase transformation of gastric cancer cells and increases cell adhesion and invasion ability. Knockdown of VWCE can significantly downregulate cell proliferation, anchors independent growth and invasion of gastric cancer cells [14]. Furthermore, the function of VWCE is closely related to signal transduction, which regulates cell adhesion, migration, and interaction [15], and promotes cancer development and progression [16]. Above these results propose that VWCE may be a novel proto-oncogene. However, the interaction between VWCE expression and immune infiltration in breast cancer remains an unsolved problem.

In the present study, we investigated the expression pattern of VWCE using several bioinformatics web servers including Tumor Immune Estimation Resource (TIMER) and DriverDB databases. Furthermore, the genes co-expressed with VWCE and gene ontology (GO) enrichment analysis were investigated by the STRING and Enrichr web servers. Next, we performed the single nucleotide variation (SNV), copy number variation (CNV), and pathway activity analysis by GSCALite. Moreover, we investigated the correlation between VWCE expression and immune infiltration level in breast cancer by TIMER database and TISIDB databases, and further verified the results using Quantitative Real-Time PCR (RT-PCR), Western blotting, and immunohistochemistry.

## Methods

### VWCE expression analysis

The expression of VWCE in different cancer types was investigated using the TIMER web tool (https://cistrome.shinyapps.io/timer/) [17] and DriverDB (http://driverdb.tms.cmu.edu.tw/) [18]. TIMER web server is a comprehensive resource for systematical analysis of immune infiltrating from diverse cancer types. DriverDB is a cancer genomics database that incorporates somatic mutation, RNA expression, miRNA expression, methylation, copy number variation, and clinical data in addition to annotation bases. This database also uses published bioinformatics algorithms to identify driver genes and present them with different molecular features [18]. The differential expression squares indicate whether the gene has significant with p-value < 0.05 and whether it is differentially expressed. In the study, we explored the expression of VWCE between different tumors and normal tissue using the above database.

### Association of clinicopathological parameters with VWCE in breast cancer patients

Bc-GenExMiner v 4.4 (http://bcgenex.centregauducheau.fr/) is a statistical mining tool of published annotated breast cancer transcriptomic data [DNA microarrays (n = 10,001) and RNA-seq (n = 4712)]. It offers the possibility to explore the gene-expression of genes of interest in breast cancer [19]. We analyzed the expression level of VWCE based on various classification parameters, such as the estrogen receptor (ER) and progesterone receptor (PR) expression, human epidermal growth factor receptor-2 (HER2) status (ER+ /ER−, PR+ /PR−, and HER2+ /HER2−), nodal status (N+ /N−), age (Y21–40, Y40–70, and Y70–97), and subtypes (basal-like, HER2-E, luminal A, luminal B, and normal basal-like).

### Functional and pathway enrichment analysis

We identified genes co-expressed with VWCE using the STRING database (https://string-db.org/) [20]. Protein name: VWCE, and organism: homo sapiens were analyzed. Enrichr, a comprehensive tool for gene enrichment analysis (http://amp.pharm.mssm.edu/Enrichr), the database integrates biological information and provides a complete set of function annotation data of genes and proteins for users to analyze [21]. In the study, we performed gene ontology (GO) enrichment analysis (biological process, molecular function, and cellular component) to identify gene interactions and provides a complete set of gene functional annotation data and proteins for users to analyze functions or signaling pathways [22].

### Gene set cancer analysis

GSCALite is a web-based analysis platform for gene set cancer analysis. With the acquisition of big data of cancer genomics, it is very useful and urgent to provide a platform for gene set analysis in cancer [23]. It offers a web-based platform for single nucleotide variation (SNV), copy number variation (CNV), methylation, and pathway activity. SNV module presents the SNV frequency and variant types of the gene set in selected cancer types. On the CNV module, the statistics of heterozygous and homozygous CNV of each cancer type are displayed as pie chat for gene set, and Pearson correlation is performed between gene expression and CNV of each gene in each cancer to help to analyze the gene expression significantly affected by CNV. Pathway activity module presents the difference of genes expression between pathway activity groups (activation and inhibition) that defined by pathway scores.

### Analysis of the correlation between VWCE expression and the immune cell infiltration

The abundance of tumor-infiltrating immune cells (TIICs) in breast cancer was predicted using the TIMER web tool (https://cistrome.shinyapps.io/timer/) and the TISIDB database (http://cis.hku.hk/TISIDB) [24]. TIMER determines the abundance of TIIC including B cells, CD8^+^ T cells, CD4^+^ T cells, macrophages, neutrophils, and dendritic cells based on the statistical analysis of gene expression profiles [25]. In this study, the Spearman correlation test of timer was used to analyze the correlation between VWCE expression and different immune cell abundance, and to study the effect of immune invasion on the prognosis of breast cancer. TISIDB is a web portal for tumor and immune system interaction, which integrates multiple heterogeneous data types. It integrates 988 reported immune-related anti-tumor genes, high-throughput screening technology, molecular biology, and paracancerous multi-component data, as well as various resources of immunological data retrieved from 7 public databases [25]. In this study, the TISIDB was used to investigate correlation between VWCE expression and lymphocytes, and immunomodulators.

### Quantitative real-time PCR

The assay was conducted in triplicate using the following primer sets: si#1 were 5’- GAGGTGTGAAGCTGCTTGTTCC, sequences of si#2 were 5'- AGACACAGACGGTGCAGTTCTC. The amplification was carried out in a 20 µl reaction system consisting of SYBR Green mixture (16.4 µL), cDNA (2 µL) and 0.8 µl forward and reverse primers (10 µM) under the following conditions: pre denaturation at 95 ℃ for 30 s, denaturation at 95 ℃ for 40 cycles for 5 s, and annealing/extension at 60 ℃ for 34 s. All reactions were repeated. RT-PCR was performed using 2 × TB Green Premix Ex Taq (Takara) in a Bio-Rad detection system. The data for each gene was normalized against the β-actin levels.

### Western blotting

Proteins were separated using 12% SDS-PAGE and were then transferred to a polyvinylidene fluoride (PVDF) membrane and probed with their respective antibodies. The T47D and SK-BR-3 cells were homogenized in RIPA lysis buffer (Meilunbio, Shanghai, China) containing protease inhibitor mixture on ice for 30 min, and centrifuged for 10 min at 12,000 rpm in a 4 ˚C Eppendorf microfuge (Thermo, USA). Loading buffer was added to the protein lysate and boiled for 5 min. The following primary antibodies were used: Anti-VWCE (1:1000; Affinity Biosciences), anti-STAT1 (1:50; Affinity Biosciences), and anti-MS4A4A (1:100; Affinity Biosciences) were used as primary antibodies. MXB was used for secondary antibody detection. The immunocomplexes were then visualized by chemiluminescent imaging.

### Immunohistochemistry

This study was performed on archived tissues from 5 diagnosed cases of breast cancer and 3 adjacent normal breast tissue cases were obtained from the Shenzhen Second People’s Hospital. This study was approved by the Ethics Committee of Shenzhen Second People’s Hospital in accordance with the principles of the Declaration of Helsinki. To validate the relationship between VWCE expression and tumor-infiltrating immune cells, we performed immunohistochemistry to assess VWCE, STAT1, and MS4A4A. First, the collected tissue samples were embedded, sliced, and dewaxed; then performed antigen repair was performed as follows: prepared 200 mL of 10 × repair solution, such as 20 mL 10 × repair solution + 180 mL distilled water was prepared, each slice was placed in the repair box and close the lid was closed, the box was placed in a 100 °C water bath and reacted for 20 min, then naturally cooled to room temperature; then, it was incubated in 3% H_2_O_2_ deionized water at room temperature in the dark for 10 min and rinsed with distilled water 2–3 times, for 3 min each time. The water around the slices was wiped off with filter paper, draw a circle of oil, and add an appropriate amount of blocking solution was added for protein blocking and the slices were incubated at room temperature for 20 min. The tissue was then incubated with the primary antibody and incubated overnight at 4 °C, and then incubated with the secondary antibody. Avidin was added, incubated at room temperature in the dark for 20 min, then washed with PBST, followed by DAB color development + hematoxylin counterstaining and dehydration. Lastly, an image was taken with a scanning microscope. All of the stained sections were assessed for the degree of immunostaining and scored by two pathologists. The expression density of VWCE, STAT1, and MS4A4A in breast cancer tissue was quantitated by scoring staining intensity, including negative (–) and weak (+) staining, moderate (++) and strong (+++) staining, respectively [26].

### Statistical analysis

The results of Kaplan–Meier plots were presented with HRs and p values from a log-rank test. Spearman correlation coefficient was used to measure the expression correlation among genes. Data were presented as the means of the results from at least three independent experiments. The data were processed using one-way analysis of variance (ANOVA), and Student’s t-test was employed to assess significant differences. A value of p < 0.05 was considered statistically significant.

## Results

### VWCE mRNA expression levels in various types of cancer

**Fig. 1:**
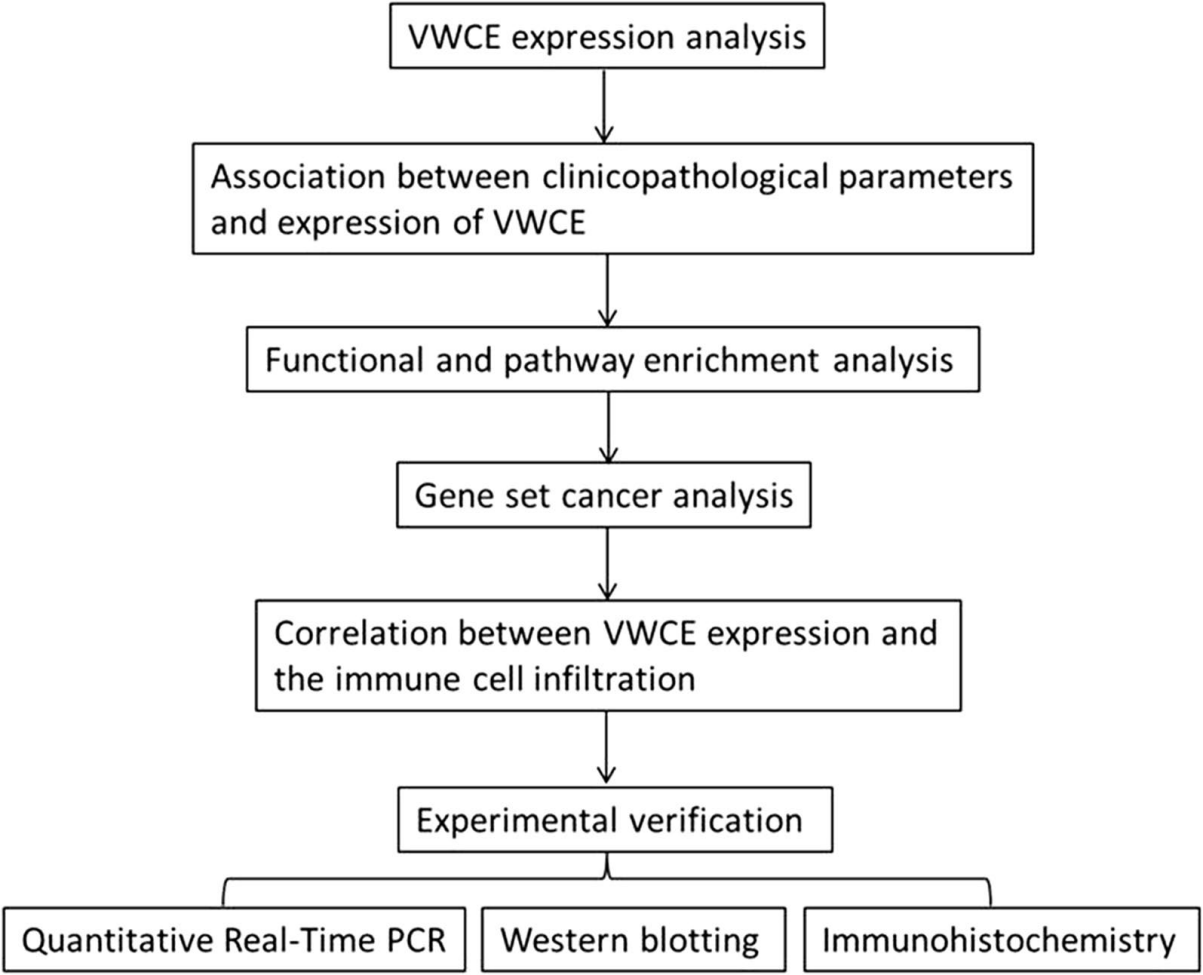
Experimental flowchart

**Fig. 2:**
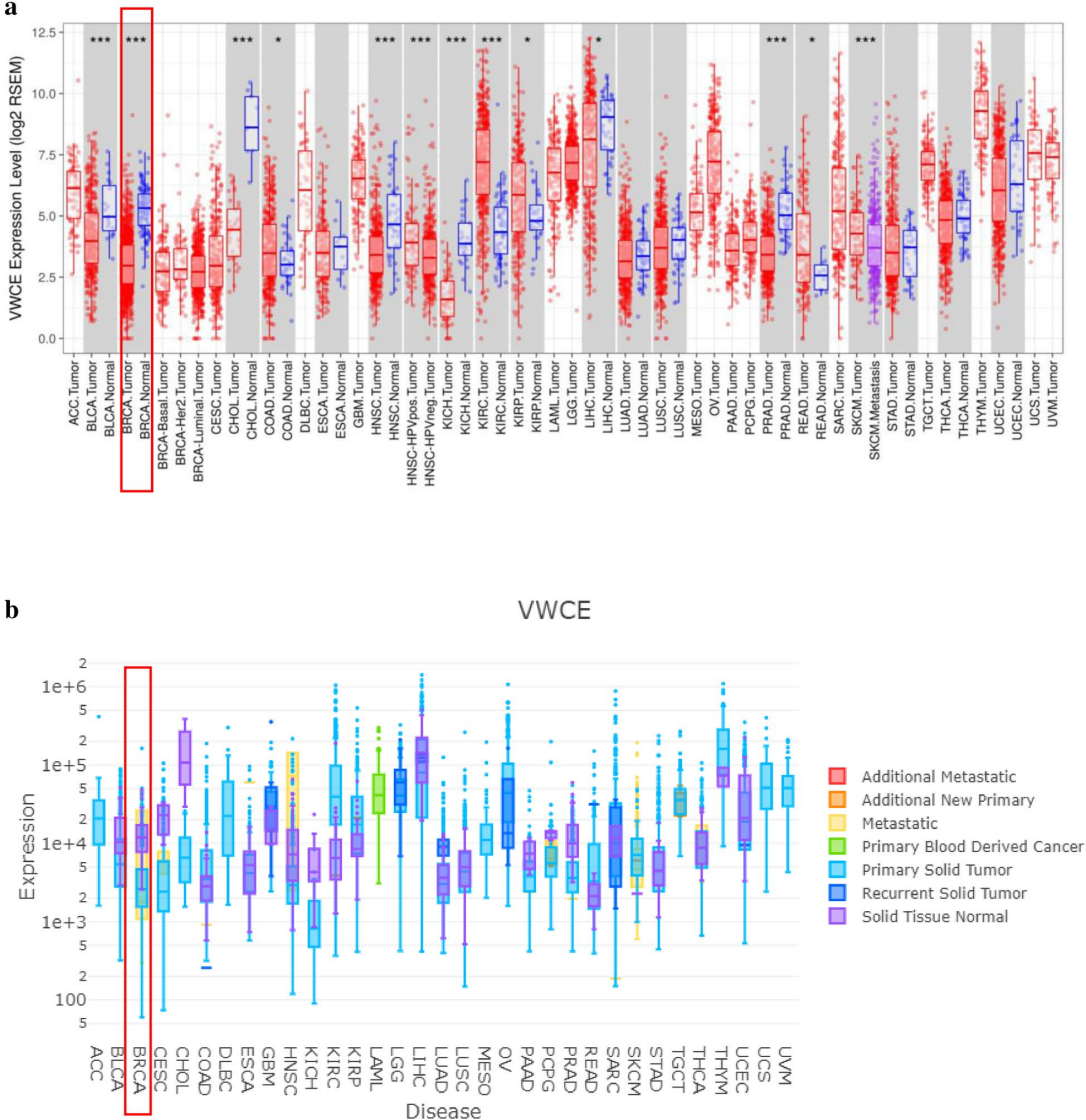
Transcription levels of VWCE in different types of cancer and normal tissues. **a** VWCE expression levels in different tumor types from the TCGA database were determined using TIMER. The color intensity (red or blue) is directly proportional to the significance level of upregulation or downregulation, respectively. VWCE mRNA expression levels in breast cancer are delineated with red highlights. P-value Significant Codes: 0 ≤ *** < 0.001 ≤ ** < 0.01 ≤ * < 0.05 ≤ . < 0.1. **b** The expression levels of VWCE expression in different tumor types using DriverDB. Red represents up-regulated genes with log2 (fold change) > 1 and green represents down-regulated genes with log2 (fold change) < − 1. Blue and purple dashed lines represent the average value of all tumor and normal tissues, respectively

We performed the expression of VWCE and its association with the prognosis and immune infiltration level in this study (Fig. 1). Information in the TIMER database uncovered that mRNA expression of VWCE was fundamentally lower in breast cancer tissues when compared with normal tissues (p < 0.01). As shown in Fig. 2a, compared with normal tissues, the VWCE transcription levels were significantly lower in BLCA (bladder urothelial carcinoma), BRCA (breast invasive carcinoma), CHOL (cholangiocarcinoma), HNSC (head and neck squamous cell carcinoma), KICH (kidney chromophobe), and PRAD (prostate adenocarcinoma). Additionally, to confirm the differences in VWCE expression in BRCA, VWCE expression was analyzed using the DriverDBv3 databases. Figure 2b also showed that VWCE mRNA expression was lower in breast tumors than normal tissues.

### Associations between VWCE expression profiles and clinicopathological parameters in breast cancer patients

**Fig. 3:**
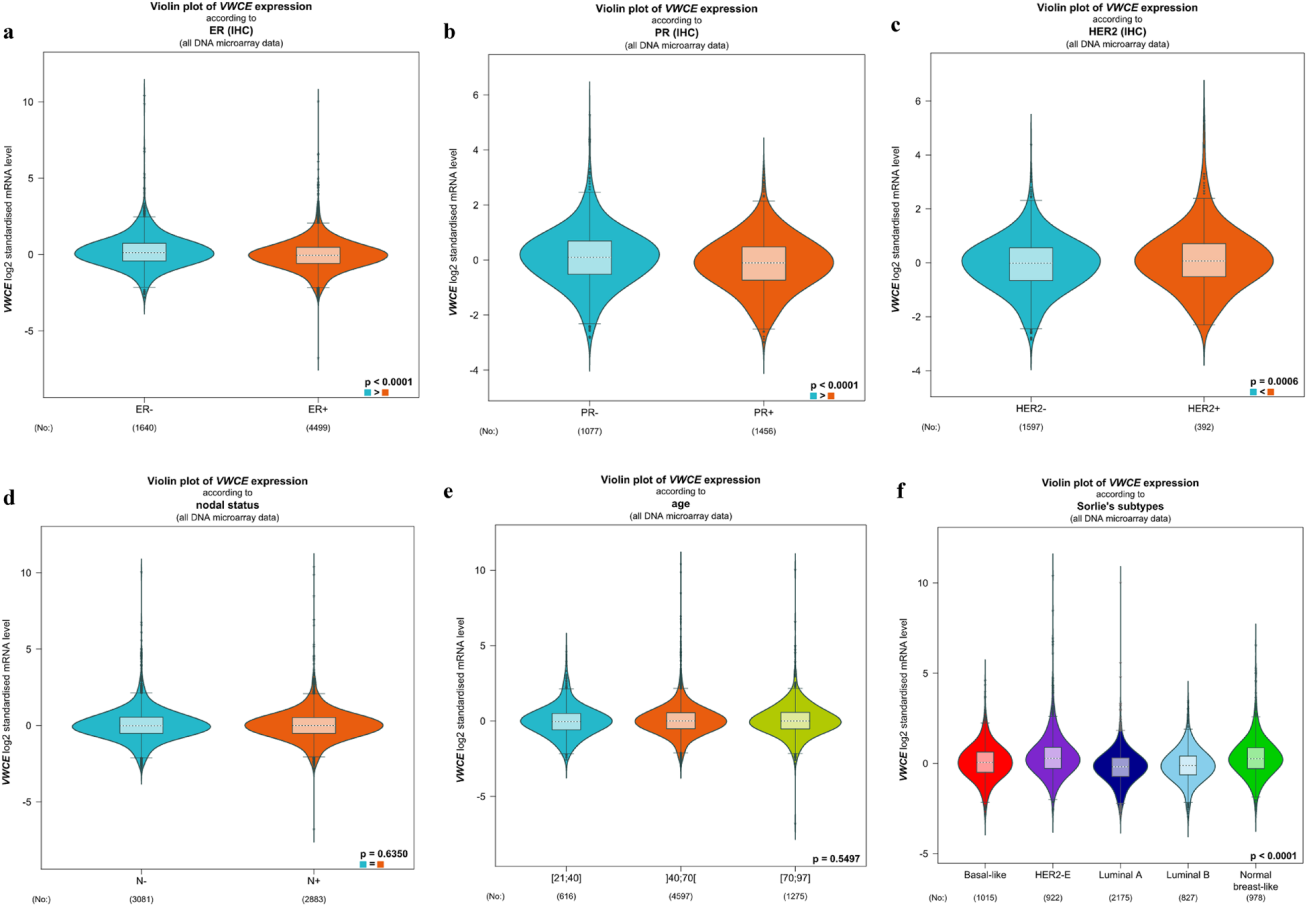
The relationship between VWCE expression and the clinical characteristics of breast cancer patients. A: ER status (ER−, ER+), B: PR status (PR−, PR +), C: HER2 status (HER2+, HER2−), D: age (> 21 ≤ 40, > 40 ≤ 70, and > 70 ≤ 97), E: nodal status (N−, N+). F: subtypes (Basal-like, HER2-E, Luminal A, Luminal B, and normal basal-like)

We investigated the relationship between VWCE expression and the clinical characteristics of breast cancer patients using bc-GenExMiner 4.4. There were remarkably differential expression levels of VWCE mRNA in ER status (ER- > ER + , p < 0.0001, Fig. 3a), PR status (PR- > PR + , p < 0.0001, Fig. 3b), and HER2 status (HER2+ > HER2−, p = 0.0006, Fig. 3c). However, no significant expression difference of VWCE mRNA was found in age and nodal status (Fig. 3d, ee). In addition, we also found that there was significant difference between the expression of VWCE and subtypes (HER2-E > basal-like, p < 0.0001; luminal A < basal-like, p < 0.0001; luminal A < HER2-E, p < 0.0001; luminal B < HER2-E, p < 0.0001) (Fig. 3f). These results suggest that VWCE expression may serve as a potential diagnostic indicator in breast cancer.

### Biological interaction network of VWCE

**Fig. 4:**
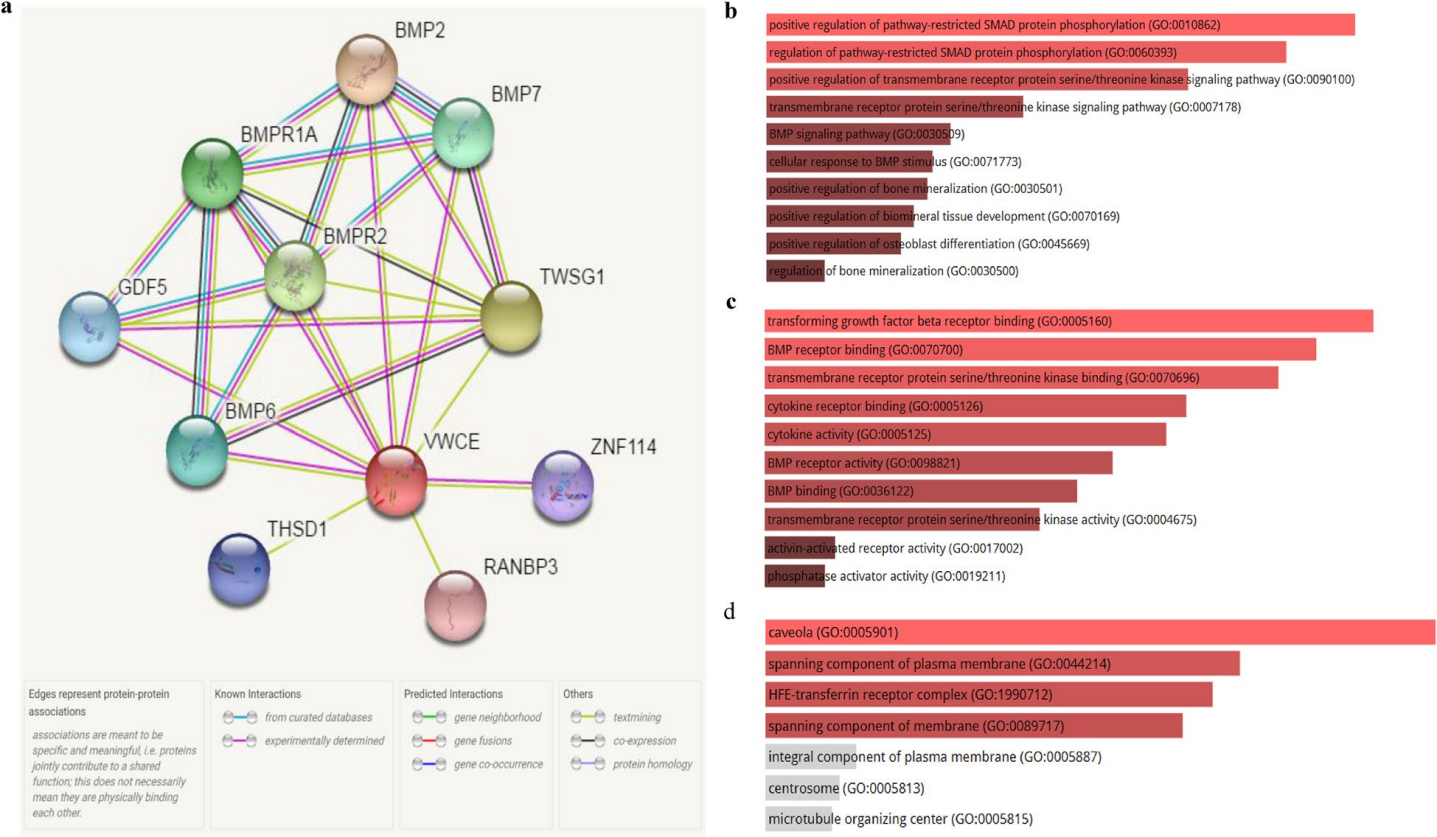
Protein interaction and enrichment analysis of VWCE. **a** The PPI network of VWCE was constructed. **b** gene ontology (GO) enrichment analysis was performed to identify gene interactions from three functional categories, i.e., **a** biological process, **b** molecular function, and **c** cellular component using Enrichr

Since this study is based on data obtained, we analyzed the functional protein association network by the STRING database. The co-expression analysis revealed that VWCE was co-expressed with bone morphogenetic protein 2 (BMP2), twisted gastrulation protein homolog 1 (TWSG1), bone morphogenetic protein receptor type-2 (BMPR2), bone morphogenetic protein receptor type-1A (BMPR1A), bone morphogenetic protein 7 (BMP7), Bone morphogenetic protein 6 (BMP6), growth/differentiation factor 5 (GDF5), thrombospondin type-1 domain-containing protein 1 (THSD1), zinc finger protein 114 (ZNF114) and ran-binding protein 3 (RANBP3), whose correlation scores were 0.758, 0.659, 0.654, 0.639, 0.628, 0.608, 0.594, 0.581, 0.546, and 0.535, respectively (Fig. 4a), suggesting that they may be functional partners in breast cancer.

To examine the function of the identified genes, biological analyses (GO enrichment and KEGG pathway analysis) were performed via the Enrichr online database (Fig. 4b, Additional file 1: Table S1). GO analysis results demonstrated that the biological processes of these proteins were mainly involved in positive regulation of pathway-restricted SMAD protein phosphorylation (GO: 0010862), positive regulation of bone mineralization (GO: 0030501), and regulation of pathway-restricted SMAD protein phosphorylation (GO: 0060393). Molecular function (MF) were mainly enhanced in BMP receptor binding (GO: 0070700), BMP receptor activity (GO: 0098821), and transmembrane receptor protein serine/threonine kinase binding (GO: 0070696). Cell component was significantly enriched in the spanning component of the plasma membrane (GO: 0044214), HFE-transferrin receptor complex (GO: 1990712), and spanning component of membrane (GO: 0089717). Therefore, we speculate that VWCE may be related to the occurrence and development of cancer.

### Gene set cancer analysis

**Fig. 5:**
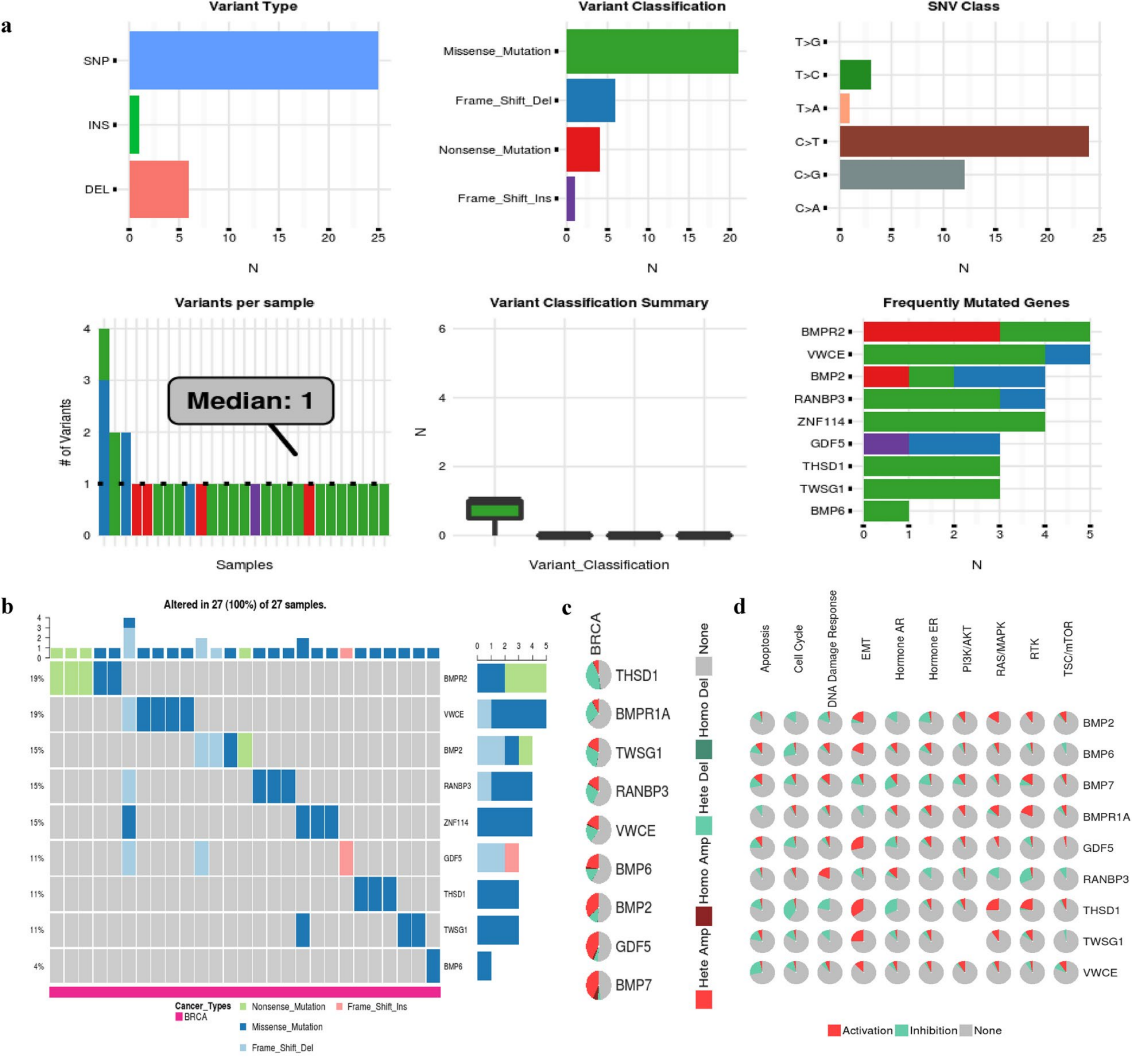
Single Nucleotide Variation (SNV), Copy Number Variation (CNV), and pathway activity analysis by GSCALite. **a** SNV frequency and variant types of VWCE and its co-expression genes in breast cancer. **b** The expression of VWCE and its co-expression genes was correlated with the activation or inhibition of multiple oncogenic pathways

To further understand the SNM, CNV, and pathway activity of these proteins, we performed the analysis by GSCALite (Fig. 5). From the SNV module, the SNV frequency of BMPR2, VWCE, BMP2, RANBP3, and ZNF114 are in the top five, which are 19, 19, 15, 15, and 15%, respectively. Among them, the variant types of VWCE, RANBP3, and ZNF114 are missense mutations and frame-shift-Del. On the CNV module, the main copy number variants of these genes include heterozygous amplification and heterozygous deletion. Thus, these data indicate that VWCE mutations are associated with its expression. Moreover, the results of pathway analyses reveal that the expression of VWCE is related to the activation or inhibition of multiple oncogenic pathways. VWCE expression mainly inhibits the apoptosis and the cell cycle pathway by THSD1 and BMP6 expression. However, the EMT pathway mainly is activated by TWSG1, THSD1, and GDF5 expression. Therefore, the biological interaction network of VWCE is engaged with protein complex formation, protein regulation, and cancer processes.

### VWCE expression impacts the prognosis and correlates with immune infiltration level in breast cancer

**Fig. 6:**
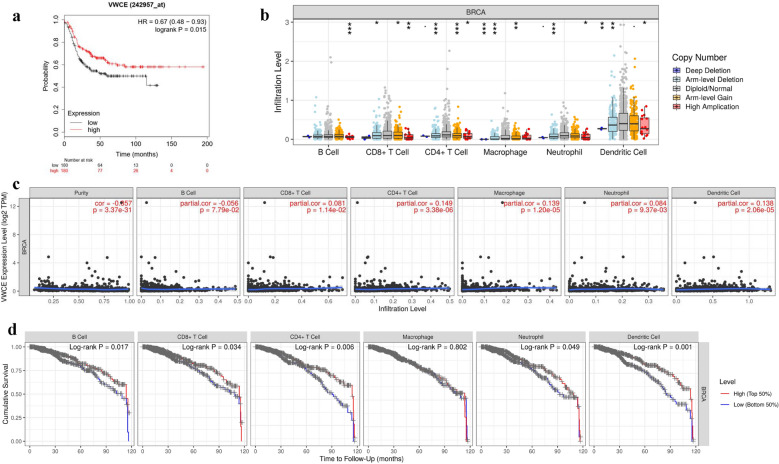
Correlation between VWCE expression and immune infiltration level in breast cancer. **a** The prognostic significance of high and low expression of VWCE in breast cancers using the Kaplan–Meier plotter database. **b** The correlation between somatic copy number alterations (SCAN) and abundance of immune infiltrates of VWCE, including deep deletion, shallow deletion, diploid/normal, low-level gain, and high amplification. Box plots are presented to show the distributions of each TIIC subset for each copy number status in breast cancer using the same statistical tests as in the ‘‘mutation’’ module. Color images are available online. **c** Correlation analysis of VWCE expression and infiltration levels of immune cells in breast cancer using the TIMER database, including B cell abundance, CD8^+^ T cells, CD4^+^ T cells, macrophages, neutrophils, and dendritic cells relative to VWCE expression. **d** Correlation between VWCE expression and breast cancer prognosis in immune infiltration

To better understand the relevance and underlying mechanisms of VWCE expression in breast cancer, we investigated the relationship between the expression of VWCE and prognosis of breast cancer patients in the Kaplan–Meier plotter database. As shown in Fig. 6a, the high expression of VWCE was associated with better prognosis (HR = 0.67, p = 0.015). These results suggest that the expression level of VWCE can impact the prognosis of breast cancer. Moreover, we analyzed the correlation between somatic copy number alterations and the abundance of immune infiltrates of VWCE (Fig. 6b). SCNA module provides the comparison of tumor infiltration levels among tumors with different somatic copy number alterations for a given gene. SCNAs are defined by GISTIC 2.0, including deep deletion (− 2), arm-level deletion (− 1), diploid/normal (0), arm-level gain (1), and high amplification (2). Box plots are presented to show the distributions of each immune subset at each copy number status in breast cancer.

Previous studies have reported that the survival time of patients in several cancers depends on the number and activity of tumor-infiltrating lymphocytes [27, 28]. Therefore, we investigated whether VWCE expression was correlated with immune infiltration levels in breast cancer. We assessed the correlation between VWCE expression and immune infiltration levels in breast cancer using the TIMER database. The results showed that the expression of VWCE was significantly correlated with tumor purity and infiltration level of CD4^+^ T cell, macrophages, and dendritic cell in breast cancer (Fig. 6c). However, the clinical impact of immune cells in breast cancer remains poorly understood. Therefore, it is necessary to conduct a more comprehensive analysis of tumor immunity to better understand. Next, we analyzed immune cells (B cell, CD8^+^ T cell, CD4^+^ T cell, macrophages, neutrophils, and dendritic cells) on the prognosis in breast cancer, the expression of these immune cells was divided into high and low levels by using the median expression. The results showed that high infiltration levels of B cell, CD8^+^ T cell, CD4^+^ T cell, neutrophils, and dendritic cell (Fig. 6d) were significantly associated with better survival (p < 0.05), whereas macrophages were not (p > 0.05), it is worth of further research and exploration. These findings strongly indicated that VWCE played an important role in immune infiltration in breast cancer.

### Regulation of immune molecules by VWCE

**Fig. 7:**
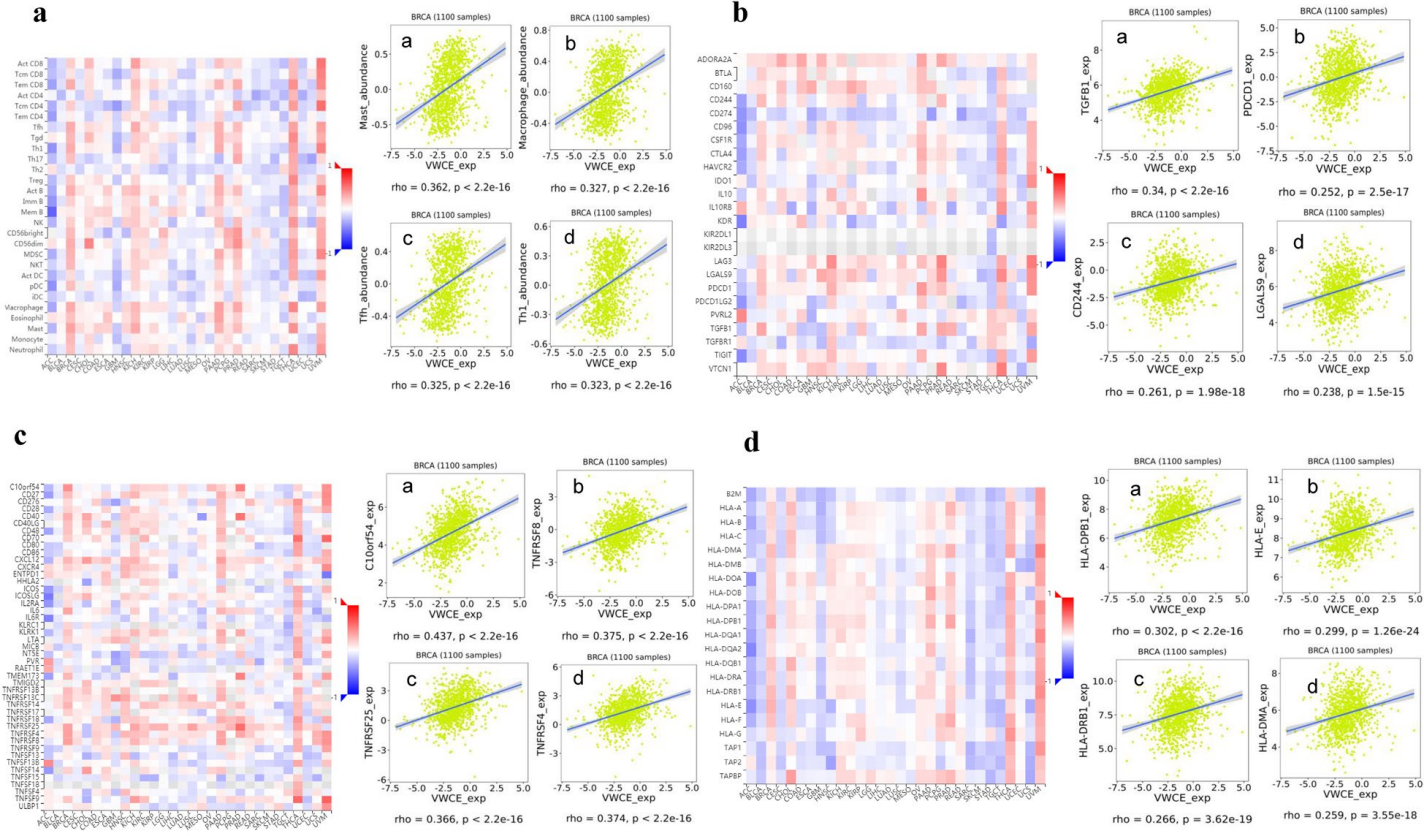
Spearman’s correlation of VWCE with lymphocytes and immunomodulators (TISIDB). A: Relations between the abundance of TILs and VWCE expression. **a**–**d** Top 4 TILs displaying the greatest correlation with VWCE expression. **b** Relations between the abundances of immunoinhibitors and VWCE expression. **a**–**d** Top 4 immunoinhibitors displaying the greatest correlation with VWCE expression. **c** Relations between abundances of immunostimulators and VWCE expression. **a**–**d** Top 4 immunostimulators displaying the greatest Spearman’s correlation with VWCE expression. **d** Relations between the abundance of MHC molecules and VWCE expression. **a**–**d** Top 4 MHC molecules displaying the greatest correlation with VWCE expression

To further study the regulation of VWCE on immune molecules in breast cancer, we used the TISIDB database to conduct an integrated analysis to predict correlations between VWCE expression and lymphocytes and immunomodulators (Fig. 7). Interestingly, we found the greatest correlation between VWCE expression and TILs included mast (Spearman: rho = 0.362, p < 2.2e−16), macrophages (Spearman: rho = 0.327, p < 2.2e−16), Tfh (Spearman: rho = 0.325, p < 2.2e−16), and Th1 (Spearman: rho = 0.323, p < 2.2e−16) (Fig. 7a). Immunomodulators can be further classified into immunoinhibitors, immunostimulators, and major histocompatibility complex (MHC) molecules [29]. The correlation between the expression level of VWCE and immunoinhibitors was showed in Fig. 7b, among these immunoinhibitors, the expression of VWCE has the strongest correlation with TGFB1 (Spearman: rho = 0.34, p < 2.2e−16), CD244 (Spearman: rho = 0.261, p = 2.5e−18), PDCD1 (Spearman: rho = 0.252, p = 2.5e−17), and LGALS9 (Spearman: rho = 0.238, p = 1.5e−15). Figure 7c shows the correlation between VWCE expression and immunostimulators, and the immunostimulators displaying the greatest correlation included C10orf54 (Spearman: rho = 0.437, p < 2.2e−16), TNFRSF8 (Spearman: rho = 0.375, p < 2.2e−16), TNFRSF4 (Spearman: rho = 0.374, p < 2.2–16), and TNFRSF25 (Spearman: rho = 0.366, p < 2.2e−16). Next, we compared the correlation between VWCE expression and MHC molecules (Fig. 7d). The MHC molecules that showed the greatest correlation included HLA-DPB1 (Spearman: rho = 0.302, p < 2.2e−16), HLA-E (Spearman: rho = 0.299, p < 1.26e−24), HLA-DRB1 (Spearman: rho = 0.266, p < 3.62e−19), and HLA-DMA (Spearman: rho = 0.259, p < 3.55e−18). Therefore, VWCE may be involved regulating the above immune molecules.

### Correlation analysis between VWCE expression and immune marker sets

**Fig. 8:**
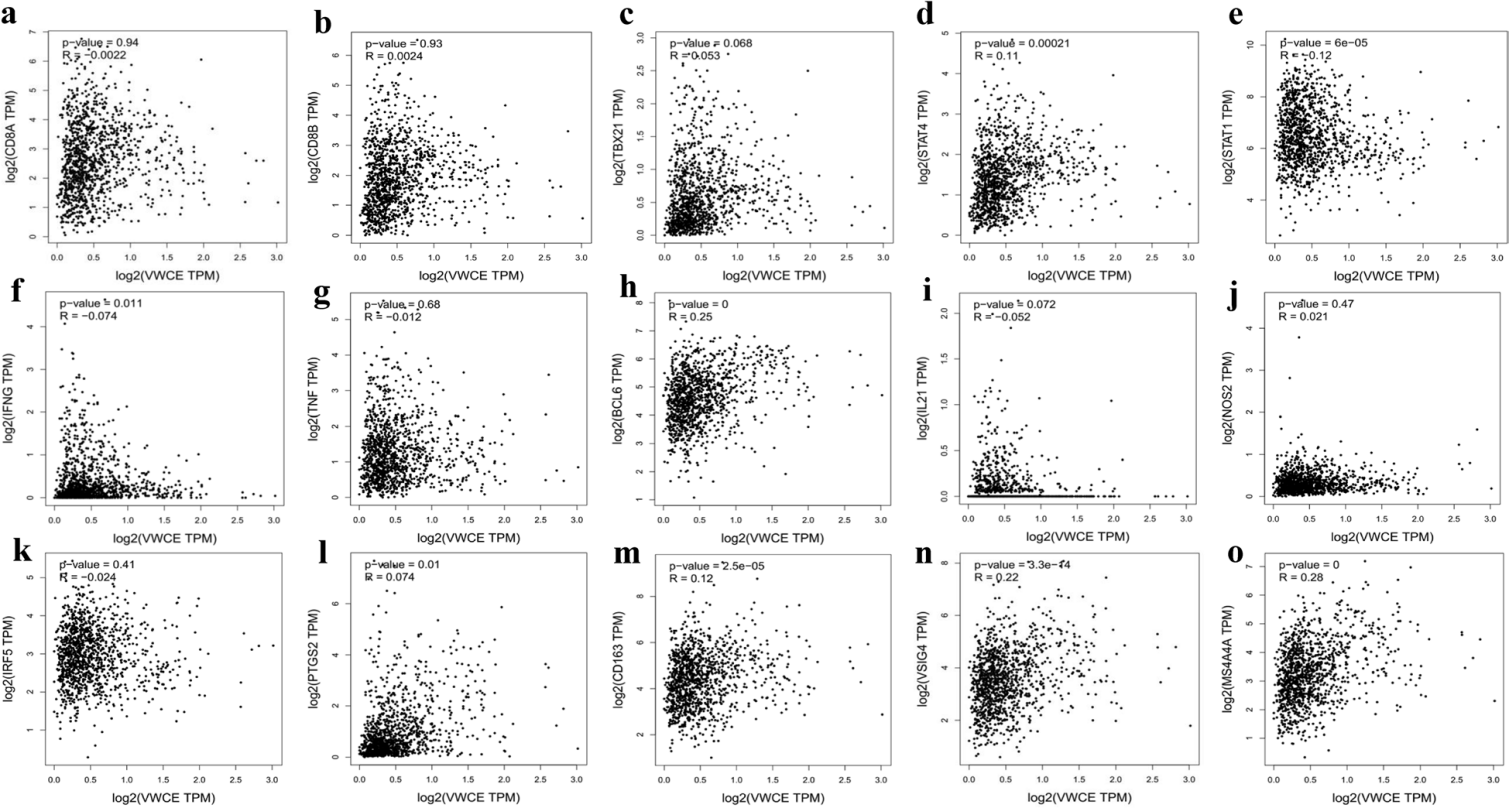
Correlation analysis of VWCE expression and the expression of marker genes of infiltrating immune cells in breast cancer using GEPIA database. Correlation of VWCE expression with various gene markers of **a**, **b**: CD8 + T cell (CD8A and CD8B), **c**–**g**: Th1(T-bet, STAT4, STAT1, IFN-γ, TNF-α), **h**, **i**: Tfh (BCL6 and IL21), **j**–**l**: M1 macrophage (INOS, IRF5, and COX2), **m**–**o**: M2 macrophage (CD163, VSIG4, and MS4A4A)

To detect the relationship between VWCE expression and various immune infiltrating cells, we focused on the correlation between VWCE expression and immune marker sets of various immune cells in BRCA using the TIMER database. The immune cells analyzed in BRCA tissues included CD8^+^ T cell, T helper 1 (Th1), follicular helper T (Tfh), M1 Macrophage, and M2 Macrophage (Table 1). The expression level of VWCE was significantly correlated with CD8^+^ T cell, Th1, M1 macrophage, and M2 macrophage. The strongest correlation was found with immune cell markers for CD8^+^ T cell (CD8B), Th1 (T-bet, STAT1), M1 macrophage (COX2), and M2 macrophage (MS4A4A). Next, we further investigated the correlation between VWCE expression and a variety of immune infiltrating cells using GEPIA database (Fig. 8; Table 2). Specifically, the expression of VWCE was significantly correlated with the expression of markers of Th1, STAT4 (r = 0.11, p = 0.00021), STAT1 (r = − 0.12, p = 6e−05), and M2 macrophage markers, CD163 (r = 0.12, p = 2.5e−05), VSIG4 (r = 0.22, p = 3.3e−14), and MS4A4A (r = 0.28, p = 0), but not with Tfh and M1 macrophage. Thus, these results suggest that VWCE may be involved in Th1 and M2 macrophage infiltrates.

**Table 1:** Correlation analysis between VWCE and relate genes and markers of immune cells using TIMER database

Description	Gene markers	None		Purity	
		Cor	P	Cor	P
CD8^+^ T cell	CD8A	0.231	7.94e−15	0.064	4.41e−02
	CD8B	0.264	4.88e−19	0.108	6.63e−04
Th1	T-bet (TBX21)	0.299	4.12e−24	0.146	3.79e−06
	STAT4	0.248	7.49e−17	0.072	2.32e−02
	STAT1	− 0.043	1.57e−01	− 0.112	3.90e−104
	IFN-γ (IFNG)	0.101	7.49e−04	− 0.038	2.31e−01
	TNF-α (TNF)	0.07	2e−02	− 0.014	6.58e−01
Tfh	BCL6	0.118	8.98e−05	0.067	3.51e−02
	IL21	0.007	8.09e−01	− 0.091	3.90e−03
M1 Macrophage	INOS (NOS2)	0.101	8.01e−04	0.08	1.21e−02
	IRF5	0.134	8.06e−06	0.059	6.36e−02
	COX2(PTGS2)	0.249	6.05e−17	0.144	5.32e−06
M2 Macrophage	CD163	0.183	1.01e−09	0.087	5.96e−03
	VSIG4	0.187	4.16e−10	0.084	8.23e−03
	MS4A4A	0.252	1.87e−17	0.136	1.71e−05

**Table 2:** Correlation between VWCE expression and immune cell infiltration in breast cancer

Description	Gene markers	Cor	p
CD8^+^ T cell	CD8A	0.0022	0.94
	CD8B	0.0024	0.93
Th1	T-bet (TBX21)	0.053	0.068
	STAT4	0.11	0.00021
	STAT1	− 0.12	6e−05
	IFN-γ (IFNG)	− 0.074	0.011
	TNF-α (TNF)	− 0.012	0.68
Tfh	BCL6	0.25	0
	IL21	− 0.052	0.072
M1 Macrophage	INOS (NOS2)	0.021	0.47
	IRF5	− 0.024	0.41
	COX2(PTGS2)	0.074	0.01
M2 Macrophage	CD163	0.12	2.5e−05
	VSIG4	0.22	3.3e−14
	MS4A4A	0.28	0

**Fig. 9:**
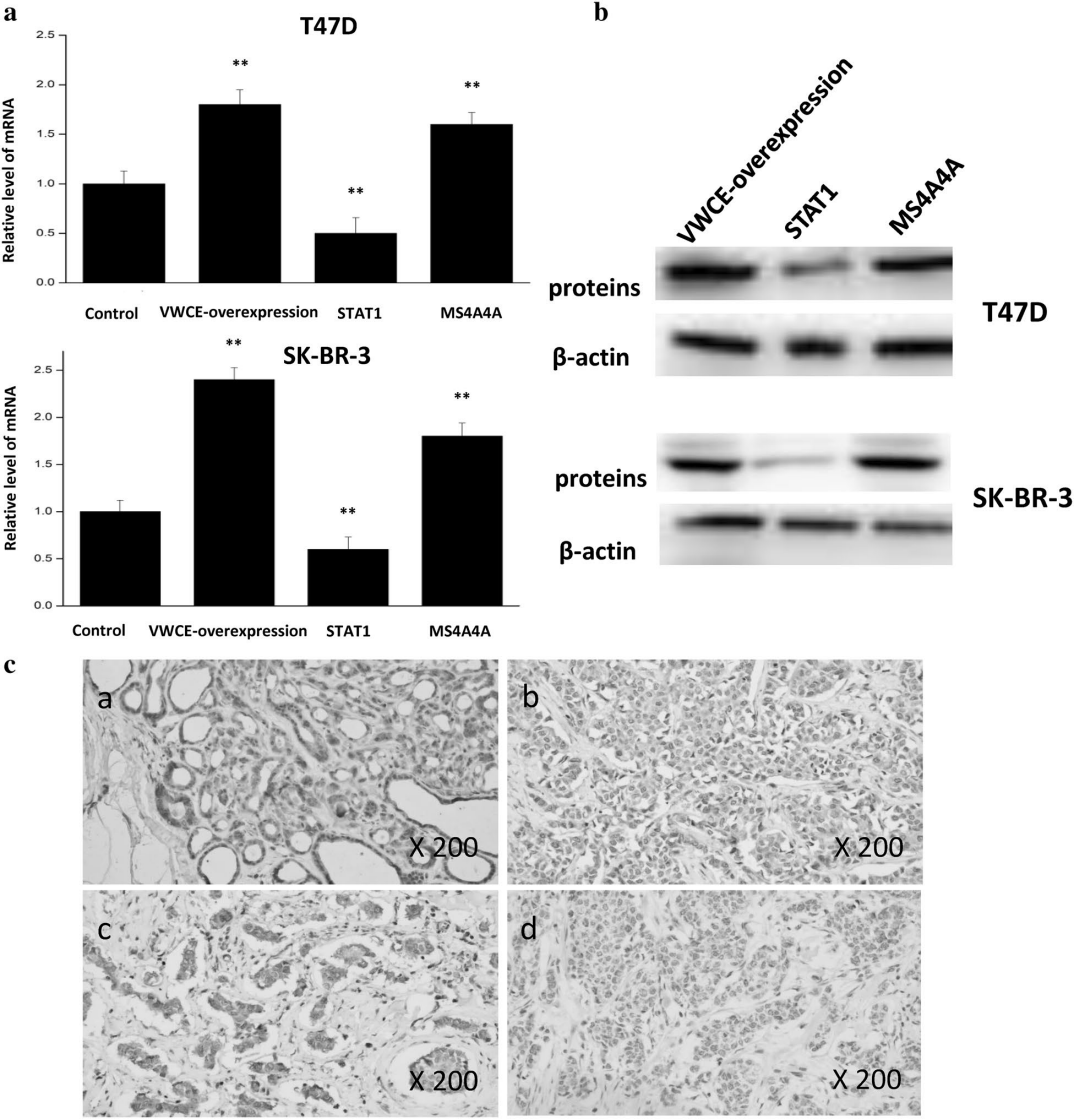
The expression of STAT1 and MS4A4A after VWCE-overexpression treatment. **a** The expression of STAT1 and MS4A4A after VWCE-overexpression was analyzed by RT-PCR. **b** The correlation between VWCE-overexpression and STAT1 and MS4A4A in T47D and SK-BR-3 breast cancer cell lines was analyzed by western blotting. **C** Correlation between VWCE expression and immune infiltration level of Th1 and M2 macrophages in breast cancer. **a** The expression of VWCE in normal tissues (++), **b** The expression of VWCE in breast cancer tissues (+), **c** The expression level of STAT1 was high in breast cancer tissues (++), **d** The expression level of MS4A4A was relatively low in breast cancer tissues (+). The expression density of VWCE, STAT1, and MS4A4A in breast cancer tissue was quantitated by scoring staining intensity, including negative (−) and weak (+) staining, moderate (++) and strong (+++) staining, respectively.

We analyzed the correlation between the expression of VWCE and STAT1 (Th1 marker) and MS4A4A (M2 macrophage marker) by RT-PCR, western blotting, and immunohistochemistry (Fig. 9). The expression of STAT1 and MS4A4A were analyzed after VWCE-overexpression treatment. The RT-PCR analysis showed that VWCE-overexpression in the T47D and SK-BR-3 cells decreased the expression of STAT1, but increased the expression of MS4A4A (Fig. 9a). We then used western blotting to study the correlation between VWCE expression and these markers in T47D and SK-BR-3 breast cancer cell lines [30, 31]. The results were consistent with RT-PCR (Fig. 9b). To further examine this results, the expression level of these proteins were quantitated by scoring staining intensity, including negative (−) and weak (+) staining, moderate (++) and strong (+++) staining, respectively (Fig. 9c). We found that VWCE mainly localized in the extracellular. VWCE showed strong expression in normal tissues, but low expression in breast cancer tissues. The expression level of STAT1 was high in breast cancer tissues, while the expression level of MS4A4A was relatively low in breast cancer tissues. The results further revealed that STAT1 was negatively correlated with the expression of VWCE, but MS4A4A was positively correlated with the expression of VWCE. Taken together, the expression of VWCE relates to infiltration levels of Th1 and M2 macrophages.

## Discussion

The purpose of this study is to predict the role of VWCE in breast cancer by using an extensive bioinformatics data mining process to predict the relationship between the expression level of VWCE and immune infiltration level of different immune cells in breast cancer. To the best of our knowledge, this report is the first to highlight the relationship between VWCE expression and immune infiltrates in breast cancer.

VWCE, a new gene upregulated by hepatitis B virus X protein, is involved in the development and progression of several tumors [32, 33]. However, the role of VWCE in breast cancer remains to be elucidated. To acquire more detailed information about the potential and regulatory mechanism of VWCE in breast cancer, we performed bioinformatics analysis of open sequencing information to guide future breast cancer research. In our previous bioinformatic analysis, we found that the expression of VWCE based on online analysis tool was lower in breast cancer. These results are not consistent with the previous studies in other tumors [13, 34, 35]. We speculate that this may be related to the role and mechanism of the same gene in different tissues and cells, that is to say, malignant tumors are highly heterogeneous. In addition, to further elucidate the molecular mechanisms of VWCE in breast cancer, we searched the biological interaction network of VWCE in breast cancer by the STRING database. We found that VWCE was co-expressed with BMP2, TWSG1, BMPR2, BMPR1A, BMP7, BMP6, GDF5, THSD1, ZNF114, and RANBP3. Previous studies have highlighted the important role of BMP2 in migration and angiogenesis [36, 37]. Besides, Neill H L O et al. revealed that BMP2/BMPR1A was linked to tumor progression in dedifferentiated liposarcomas [38]. BMPER and TWSG1 are two extracellular BMP modulators, they can determine endothelial cell fate via activation of synergistic BMP and notch signaling [39]. THSD1 was proved as a novel candidate tumor suppressor gene for esophageal squamous cell carcinoma [40]. Therefore, the biological analysis results of VWCE will help researchers to better understand molecular mechanism of VWCE in breast cancer.

VWCE is composed of five chordin-like cysteine repeats and a C-type lectin domain. Chordin is a regulator of bone morphogenetic proteins (BMPs) and contains four cysteine-rich repeats, which are also called von Willebrand factor C (VWC) domains. These domains have been shown to interact with cell adhesion, migration, and the cellular matrix, indicating an important role in cancer cell EMT. For example, VWC has been shown to bind to members of transforming growth factor-β (TGF-β) superfamily and has been proposed to regulate growth factor signaling [41]. Furthermore, Du R et al. reported that VWCE promoted gastric cancer growth and invasion by activation of the β-catenin signaling pathway [14]. To explore the potential mechanism of VWCE in breast cancer, we analyzed the functional protein association network. Our study found that the molecular function of VWCE was mainly enhanced in BMP receptor binding, BMP receptor activity, and transmembrane receptor protein serine/threonine kinase binding. Moreover, previous studies have demonstrated that downregulation suppresses proliferation, invasion, and β-catenin expression in non-small cell lung cancer cells [35]. Our results suggest that the expression of VWCE is correlated with the activation or inhibition of multiple oncogenic pathways. Notably, we found VWCE expression mainly inhibited the apoptosis pathway. The prognostic analysis demonstrates that the high expression of VWCE indicates better prognosis in breast cancer. Therefore, we speculate that VWCE may be closely related to the occurrence and development of breast cancer.

Another important aspect of this study is that VWCE expression is associated with the immune infiltration levels in breast cancer. Some studies have reported that tumor-infiltrating lymphocytes were currently considered to be biomarkers highly associated with breast cancer. Generally, it is recognized that the infiltration of tumor-infiltrating lymphocytes was related to the prognosis in breast cancer, and that adjuvant treatment is relatively effective [42–44]. However, the relationship between VWCE and immune infiltration in breast cancer-luminal remains unclear. Therefore, we hypothesized that VWCE expression is associated with immune infiltration in breast cancer, and is a potential marker of the tumor immune microenvironment. The presence of macrophages is associated with reduced survival in most cancers [45]. Furthermore, as a potential cancer therapy, dendritic cell immunotherapy has a great prospect in animal research [46]. To the best of our knowledge, this study is the first to evaluate the association between VWCE expression and the levels of immune infiltration in breast cancer subtypes, especially in breast cancer-luminal in our immune infiltrates study, we observed that VWCE expression significantly correlates with tumor purity and significant correlations with infiltration levels of Th1 and M2 macrophage. This suggests that VWCE may activate or inhibit immune cells infiltrating the tumor, although the underlying mechanism involved is unknown. Macrophages are a group of differentiated immune cells and classify as M1 macrophages and M2 macrophages [47]. They play an important role in development, homeostasis, and immunity [48].

Recent studies have shown that the ability of STAT1 to inhibit STAT4 activation prevents the development of Th1 responses [49]. In addition, studies have shown that most M2 macrophages are considered to be tumor-associated macrophages, which can promote tumor angiogenesis, immunosuppression, and metastasis [50, 51]. CD163 is considered to be a marker of M2 macrophage [52]. CD163 positive cancer cells were significantly associated with shorter progression-free survival and lower overall survival [53]. MS4A4A is a novel cell surface marker for M2 macrophages, which is essential for dectin-1-dependent activation of NK cell-mediated anti-metastatic properties. Hence, our study suggests that VWCE serve as a potential biomarker is associated with clinical pathologic features and immune infiltrates in breast cancer.

To further study the regulation of VWCE on immune molecules in breast cancer, we conducted an integrated analysis to predict correlation between VWCE expression and some immunoinhibitors, immunostimulators, and MHC molecules using TISIDB database. Our results demonstrated a positive correlation between VWCE expression and lymphocytes (such as mast, macrophages, Tfh, and Th1), immunoinhibitors (such as TGFB1, CD244, PDCD1, and LGALS9), immunostimulators (such as C10orf54, TNFRSF8, TNFRSF4, and TNFRSF25), and MHC molecules (such as HLA-DPB1, HLA-E, HLA-DRB1, and HLA-DMA). TGFB1 is a regulatory cytokine that can inhibit and promote breast cancer cell lines and tissue. High serum TGFβ1 level predicts better survival in breast cancer [54]. A previous study showed that macrophages play an important role in development, homeostasis, and immunity. Besides, TNFRSF was demonstrated playing crucial roles in both innate and adaptive immunity [55]. Interestingly, we found VWCE expression was significantly correlated with T cell exhaustion markers such as PDCD1. The PD1/PDL1 axis was proved a promising therapeutic target in aggressive breast cancers, and mainly regulated the function of tumor cells and TILs [56]. Therefore, VWCE associated with these immune molecules, may provide a new target for studying the immune evasion of breast cancer cells, and can potentially serve as an immunotherapeutic target for breast cancer. In addition, VWCE expression was negatively correlated with the expression of STAT1 (Th1 marker, r = − 0.12, p = 6e−05), but positively correlated with the expression of MS4A4A (r = 0.28, p = 0). We further verified these markers of Th1 and M2 macrophage by RT-PCR, western blotting, and immunohistochemistry, these results suggested that VWCE may be involved in Th1 and M2 macrophage infiltrates. This work has taken some important first steps in this direction. However, there are some problems to be solved in the future work [57].

Since this study is based on data obtained from publicly available databases, there are some limitations. The data we collected in this study lacked some information and required large samples to reliably interpret the data. Also, the mechanism of VWCE regulating the infiltration of immune cells is required for further study (Additional file 2).

## Conclusion

VWCE may be a potential biomarker and is associated with immune infiltration of breast cancer, suggesting VWCE as a therapeutic target to modulate the anti-tumor immune response. All in all, a comprehensive understanding the correlation between VWCE expression of prognosis and immune infiltration will offer new insights for immunotherapy of breast cancer.

## Supplementary Information

**Additional file 1**: **Table S1**. GO enrichment and KEGG pathway analysis.

**Additional file 2.** The original data.

## Data Availability

The authors are grateful to freely available from TIMER (https://cistrome.shinyapps.io/timer/), DriverDB (http://driverdb.tms.cmu.edu.tw/), bc-GenExMiner v4.4 (http://bcgenex.centregauducheau.fr/), STRING database (https://string-db.org/), and Enrichr (http://amp.pharm.mssm.edu/Enrichr), and GEPIA database (http://gepia.cancer-pku.cn/).

## Abbreviations

VWCE: Von Willebrand Factor C and EGF Domains
TIMER: The tumor immune estimation resource
GO: Gene ontology
SNV: Single nucleotide variation
CNV: Copy number variation
RT-PCR: Quantitative Real-Time PCR
ER: Estrogen receptor
PR: Progesterone receptor
HER2: Human epidermal growth factor receptor-2
TNM: Tumor node metastasis
TILs: Tumor-infltrating lymphocytes
TIICs: Tumor-infiltrating immune cells
BLCA: Bladder urothelial carcinoma
BRCA: Breast invasive carcinoma
CHOL: Cholangiocarcinoma
HNSC: Head and neck squamous cell carcinoma
KICH: Kidney chromophobe
PRAD: Prostate adenocarcinoma
BMP2: Bone morphogenetic protein 2
TWSG1: Twisted gastrulation protein homolog 1
BMPR2: Bone morphogenetic protein receptor type-2
BMPR1A: Bone morphogenetic protein receptor type-1A
BMP7: Bone morphogenetic protein 7
BMP6: Bone morphogenetic protein 6
GDF5: Growth/differentiation factor 5
THSD1: Thrombospondin type-1 domain-containing protein 1
ZNF114: Zinc finger protein 114
RANBP3: Ran-binding protein 3
MHC: Major histocompatibility complex
Th1: T helper 1
Tfh: Follicular helper T
TGF-β: Transforming growth factor-β

## References

[1] Huo Q, Li Z, Cheng L, Yang F, Xie N (2020). SIRT7 is a prognostic biomarker associated with immune infiltration in luminal breast cancer. Front Oncol..

[2] Siegel RL, Miller KD, Jemal A (2019). Cancer statistics, 2019. CA Cancer J Clin.

[3] Shreedhara AK, Shanbhag S, Joseph RC (2020). A study of maternal breast feeding issues during early postnatal days. SciMed J.

[4] Qiu N, He Y, Zhang S, Hu X, Chen M, Li H (2018). Cullin 7 is a predictor of poor prognosis in breast cancer patients and is involved in the proliferation and invasion of breast cancer cells by regulating the cell cycle and microtubule stability. Oncol Rep.

[5] Baxevanis CN, Sofopoulos M, Fortis SP, Perez SA (2019). The role of immune infiltrates as prognostic biomarkers in patients with breast cancer. Cancer Immunol Immunother.

[6] Bremnes RM, Busund L-T, Kilvær TL, Andersen S, Richardsen E, Paulsen EE, Hald S, Khanehkenari MR, Cooper WA, Kao SC (2016). The role of tumor-infiltrating lymphocytes in development, progression, and prognosis of non–small cell lung cancer. J Thorac Oncol.

[7] Zhang AW, McPherson A, Milne K, Kroeger DR, Hamilton PT, Miranda A, Funnell T, Little N, de Souza CPE, Laan S (2018). Interfaces of malignant and immunologic clonal dynamics in ovarian cancer. Cell.

[8] Puccetti L, Manetti R, Parronchi P, Piccinni MP, Mavilia C, Carini M, Romagnani S, Maggi E (2002). Role of low nuclear grading of renal carcinoma cells in the functional profile of tumor-infiltrating T cells. Int J Cancer.

[9] Lee N, Zakka LR, Mihm MC, Schatton T (2016). Tumour-infiltrating lymphocytes in melanoma prognosis and cancer immunotherapy. Pathology.

[10] Gil M, Kim KE (2019). Interleukin-18 is a prognostic biomarker correlated with CD8+ T cell and natural killer cell infiltration in skin cutaneous melanoma. J Clin Med.

[11] Stanton SE, Disis ML (2016). Clinical significance of tumor-infiltrating lymphocytes in breast cancer. J Immunother Cancer.

[12] Lian Z, Liu J, Li L, Li X, Tufan NL, Clayton M, Wu MC, Wang HY, Arbuthnot P, Kew M (2003). Upregulated expression of a unique gene by hepatitis B x antigen promotes hepatocellular growth and tumorigenesis. Neoplasia.

[13] Peng W, Zhang J, Liu J (2014). URG11 predicts poor prognosis of pancreatic cancer by enhancing epithelial–mesenchymal transition-driven invasion. Med Oncol.

[14] Du R, Xia L, Sun S, Lian Z, Zou X, Gao J, Xie H, Fan R, Song J, Li X (2010). URG11 promotes gastric cancer growth and invasion by activation of β-catenin signalling pathway. J Cell Mol Med.

[15] Fan R, Li X, Du W, Zou X, Du R, Zhao L, Luo G, Mo P, Xia L, Pan Y (2011). Adenoviral-mediated RNA interference targeting URG11 inhibits growth of human hepatocellular carcinoma. Int J Cancer.

[16] Sun C, Zhang G, Cheng S, Qian H, Li D, Liu M (2019). URG11 promotes proliferation and induced apoptosis of LNCaP cells. Int J Mol Med.

[17] Li T, Fan J, Wang B, Traugh N, Chen Q, Liu JS, Li B, Liu XS (2017). TIMER: a web server for comprehensive analysis of tumor-infiltrating immune cells. Cancer Res.

[18] Liu SH, Shen PC, Chen CY, Hsu AN, Cho YC, Lai YL, Chen FH, Li CY, Wang SC, Chen M (2020). DriverDBv3: a multi-omics database for cancer driver gene research. Nucleic Acids Res.

[19] Jézéquel P, Campone M, Gouraud W, Guérin-Charbonnel C, Leux C, Ricolleau G, Campion L (2012). bc-GenExMiner: an easy-to-use online platform for gene prognostic analyses in breast cancer. Breast Cancer Res Treat.

[20] Crosara KTB, Moffa EB, Xiao Y, Siqueira WL (2018). Merging in-silico and in vitro salivary protein complex partners using the STRING database: a tutorial. J Proteomics.

[21] Kuleshov MV, Jones MR, Rouillard AD, Fernandez NF, Duan Q, Wang Z, Koplev S, Jenkins SL, Jagodnik KM, Lachmann A (2016). Enrichr: a comprehensive gene set enrichment analysis web server 2016 update. Nucleic Acids Res.

[22] Gaudet P, Škunca N, Hu JC, Dessimoz C (2017). Primer on the gene ontology. Methods Mol Biol.

[23] Akbani R, Ng PK, Werner HM, Shahmoradgoli M, Zhang F, Ju Z, Liu W, Yang JY, Yoshihara K, Li J (2014). A pan-cancer proteomic perspective on The Cancer Genome Atlas. Nat Commun.

[24] Ru B, Wong CN, Tong Y, Zhong JY, Zhong SSW, Wu WC, Chu KC, Wong CY, Lau CY, Chen I (2019). TISIDB: an integrated repository portal for tumor–immune system interactions. Bioinformatics.

[25] Li B, Severson E, Pignon JC, Zhao H, Li T, Novak J, Jiang P, Shen H, Aster JC, Rodig S (2016). Comprehensive analyses of tumor immunity: implications for cancer immunotherapy. Genome Biol.

[26] Zhang Y, Yuan Y, Liang P, Zhang Z, Guo X, Xia L, Zhao Y, Shu XS, Sun S, Ying Y (2017). Overexpression of a novel candidate oncogene KIF14 correlates with tumor progression and poor prognosis in prostate cancer. Oncotarget.

[27] Gu Y, Li X, Bi Y, Zheng Y, Wang J, Li X, Huang Z, Chen L, Huang Y, Huang Y (2020). CCL14 is a prognostic biomarker and correlates with immune infiltrates in hepatocellular carcinoma. Aging (Albany NY).

[28] Ohtani H (2007). Focus on TILs: prognostic significance of tumor infiltrating lymphocytes in human colorectal cancer. Cancer Immun..

[29] Gou R, Zhu L, Zheng M, Guo Q, Hu Y, Li X, Liu J, Lin B (2019). Annexin A8 can serve as potential prognostic biomarker and therapeutic target for ovarian cancer: based on the comprehensive analysis of Annexins. J Transl Med.

[30] Habibeh Z (2019). Effects of salvia officinalis extract on the breast cancer cell line. SciMedicine Journal.

[31] Gomez A, Santana PC, Mouro AP (2020). Dosimetry study in head and neck of anthropomorphic phantoms in computed tomography scans. SciMed J.

[32] Zou X, Li X, Liu J, Lian Z, Fan R, Du R, Xie H, Song J, Fan D (2006). Preparation and characterization of a specific monoclonal antibody against a new gene product: URG11. Hybridoma (Larchmt).

[33] Pan B, Ye Y, Liu H, Zhen J, Zhou H, Li Y, Qu L, Wu Y, Zeng C, Zhong W (2018). URG11 regulates prostate cancer cell proliferation, migration, and invasion. Biomed Res Int.

[34] Tong GD, Zhang X, Zhou DQ, Wei CS, He JS, Xiao CL, Liu XL, Zheng YJ, Chen SN, Tang HH (2014). Efficacy of early treatment on 52 patients with preneoplastic hepatitis B virus-associated hepatocellular carcinoma by compound Phyllanthus Urinaria L. Chin J Integr Med.

[35] Liu Z-L, Wu J, Wang L-X, Yang J-F, Xiao G-M, Sun H-P, Chen Y-J (2016). Knockdown of upregulated gene 11 (URG11) inhibits proliferation, invasion, and β-catenin expression in non-small cell lung cancer cells. Oncol Res Featur Preclin Clin Cancer Ther..

[36] Song X, Liu S, Qu X, Hu Y, Zhang X, Wang T, Wei F (2011). BMP2 and VEGF promote angiogenesis but retard terminal differentiation of osteoblasts in bone regeneration by up-regulating Id1. Acta Biochim Biophys Sin (Shanghai).

[37] Song J, McColl J, Camp E, Kennerley N, Mok GF, McCormick D, Grocott T, Wheeler GN, Munsterberg AE (2014). Smad1 transcription factor integrates BMP2 and Wnt3a signals in migrating cardiac progenitor cells. Proc Natl Acad Sci USA.

[38] O’Neill HL, Cassidy AP, Harris OB, Cassidy JW (2016). BMP2/BMPR1A is linked to tumour progression in dedifferentiated liposarcomas. PeerJ.

[39] Osmanagic-Myers S, Rezniczek GA (2018). Arteriovenous specification: BMPER and TWSG1 determine endothelial cell fate via activation of synergistic BMP and Notch signaling. FEBS J.

[40] Josephine KO, Lung ML, Chan PL, et al. Functional complementation and gene expression profile analysis identified THSD1 at 13q14 as a novel candidate tumor suppressor gene for esophageal squamous cell carcinoma doboku gakkai ronbunshuu f, 2008; 64(3):261–271. : 10.2208/jscejf.64.261.

[41] Christian S, Ahorn H, Koehler A, Eisenhaber F, Rodi HP, Garin-Chesa P, Park JE, Rettig WJ, Lenter MC (2001). Molecular cloning and characterization of endosialin, a C-type lectin-like cell surface receptor of tumor endothelium. J Biol Chem.

[42] Oda K, Kato K, Nakamura M, Jotatsu T, Noguchi S, Kawanami T, Kido T, Yatera K (2018). Surface marker profiles on lung lymphocytes may predict the mechanism of immune-mediated pneumonitis triggered by tumor-infiltrating lymphocytes in lung cancer patients treated with pembrolizumab. Lung Cancer.

[43] Shimizu S, Hiratsuka H, Koike K, Tsuchihashi K, Sonoda T, Ogi K, Miyakawa A, Kobayashi J, Kaneko T, Igarashi T (2019). Tumor-infiltrating CD8+ T cell density is an independent prognostic marker for oral squamous cell carcinoma. Cancer Med.

[44] Pruneri G, Gray KP, Vingiani A, Viale G, Curigliano G, Criscitiello C, Lang I, Ruhstaller T, Gianni L, Goldhirsch A (2016). Tumor-infiltrating lymphocytes (TILs) are a powerful prognostic marker in patients with triple-negative breast cancer enrolled in the IBCSG phase III randomized clinical trial 22–00. Breast Cancer Res Treat.

[45] Nielsen SR, Schmid MC (2017). Macrophages as key drivers of cancer progression and metastasis. Mediators Inflamm.

[46] Cranmer LD, Trevor KT, Hersh EM (2004). Clinical applications of dendritic cell vaccination in the treatment of cancer. Cancer Immunol Immunother.

[47] Jayasingam SD, Citartan M, Thang TH, Mat Zin AA, Ang KC, Chng ES (2019). Evaluating the polarization of tumor-associated macrophages into M1 and M2 phenotypes in human cancer tissue: technicalities and challenges in routine clinical practice. Front Oncol..

[48] Wynn TA, Chawla A, Pollard JW (2013). Macrophage biology in development, homeostasis and disease. Nature.

[49] Lieberman LA, Banica M, Reiner SL, Hunter CA (2004). STAT1 plays a critical role in the regulation of antimicrobial effector mechanisms, but not in the development of Th1-type responses during toxoplasmosis. J Immunol.

[50] Sica A, Allavena P, Mantovani A (2008). Cancer related inflammation: the macrophage connection. Cancer Lett.

[51] Shiraishi D, Fujiwara Y, Horlad H, Saito Y, Iriki T, Tsuboki J, Cheng P, Nakagata N, Mizuta H, Bekki H (2018). CD163 is required for protumoral activation of macrophages in human and murine sarcoma. Can Res.

[52] Alvarado-Vazquez PA, Bernal L, Paige CA, Grosick RL, Moracho Vilrriales C, Ferreira DW, Ulecia-Moron C, Romero-Sandoval EA (2017). Macrophage-specific nanotechnology-driven CD163 overexpression in human macrophages results in an M2 phenotype under inflammatory conditions. Immunobiology.

[53] Ma C, Horlad H, Ohnishi K, Nakagawa T, Yamada S, Kitada S, Motoshima T, Kamba T, Nakayama T, Fujimoto N (2018). CD163-positive cancer cells are potentially associated with high malignant potential in clear cell renal cell carcinoma. Med Mol Morphol.

[54] Zhou YT, Zheng LY, Wang YJ, Yang L, Xie YT, Panda I, Tian XX, Fang WG (2020). Effect of functional variant rs11466313 on breast cancer susceptibility and TGFB1 promoter activity. Breast Cancer Res Treat.

[55] Dostert C, Grusdat M, Letellier E, Brenner D (2019). The TNF family of ligands and receptors: communication modules in the immune system and beyond. Physiol Rev.

[56] Fang J, Chen F, Liu D, Gu F, Chen Z, Wang Y (2020). Prognostic value of immune checkpoint molecules in breast cancer. Biosci Rep..

[57] Kosvyra A, Maramis C, Chouvarda I (2019). Developing an integrated genomic profile for cancer patients with the use of NGS data. Emerg Sci J.

