# Supplementary material for: VWCE as a potential biomarker associated with immune infiltrates in breast cancer
Source: Cancer Cell Int. 2021 May 21;21:272. doi: 10.1186/s12935-021-01955-3 (PMC8140436; doi:10.1186/s12935-021-01955-3)
Supplement: Supplementary file 1 — Additional file 1: Table S1. GO enrichment and KEGG pathway analysis. [file 12935_2021_1955_MOESM1_ESM.doc]

**Supplementary table GO enrichment and KEGG pathway analysis**

|  | **Index** | **Name** | **Adjusted p-value** | **Odds Ratio** | **Combined score** |
| --- | --- | --- | --- | --- | --- |
| biological process | 1 | positive regulation of pathway-restricted SMAD protein phosphorylation (GO:0010862) | 8.118e-17 | 4.143e-13 | 270.79 |
|  | 2 | positive regulation of bone mineralization (GO:0030501) | 2.925e-12 | 2.132e-9 | 293.26 |
|  | 3 | regulation of pathway-restricted SMAD protein phosphorylation (GO:0060393) | 4.395e-16 | 1.121e-12 | 215.72 |
|  | 4 | regulation of aldosterone biosynthetic process (GO:0032347) | 0.000004120 | 0.0009141 | 606.06 |
|  | 5 | positive regulation of biomineral tissue development (GO:0070169) | 4.084e-12 | 2.605e-9 | 275.48 |
|  | 6 | positive regulation of osteoblast differentiation (GO:0045669) | 5.583e-12 | 3.166e-9 | 259.74 |
|  | 7 | mitral valve development (GO:0003174) | 0.000005766 | 0.001226 | 519.48 |
|  | 8 | pharyngeal arch artery morphogenesis (GO:0061626) | 0.000005766 | 0.001177 | 519.48 |
|  | 9 | atrioventricular valve morphogenesis (GO:0003181) | 5.610e-8 | 0.00001789 | 363.64 |
|  | 10 | mesenchyme development (GO:0060485) | 6.903e-8 | 0.00002072 | 340.91 |
| molecular function | 1 | BMP receptor binding (GO:0070700) | 2.715e-8 | 0.00001563 | 454.55 |
|  | 2 | BMP receptor activity (GO:0098821) | 0.000004120 | 0.0007903 | 606.06 |
|  | 3 | transmembrane receptor protein serine/threonine kinase binding (GO:0070696) | 6.903e-8 | 0.00002649 | 340.91 |
|  | 4 | BMP binding (GO:0036122) | 0.000009879 | 0.001624 | 404.04 |
|  | 5 | transforming growth factor beta receptor binding (GO:0005160) | 6.646e-9 | 0.000007649 | 165.29 |
|  | 6 | transmembrane receptor protein serine/threonine kinase activity (GO:0004675) | 0.00002493 | 0.003588 | 259.74 |
|  | 7 | activin-activated receptor activity (GO:0017002) | 0.003844 | 0.4916 | 259.74 |
|  | 8 | phosphatase activator activity (GO:0019211) | 0.004940 | 0.5686 | 202.02 |
|  | 9 | cytokine receptor binding (GO:0005126) | 6.698e-7 | 0.0001927 | 53.09 |
|  | 10 | cytokine activity (GO:0005125) | 0.000001098 | 0.0002527 | 46.92 |
| cellular component | 1 | spanning component of plasma membrane (GO:0044214) | 0.003296 | 0.7350 | 303.03 |
|  | 2 | HFE-transferrin receptor complex (GO:1990712) | 0.004392 | 0.6530 | 227.27 |
|  | 3 | spanning component of membrane (GO:0089717) | 0.006035 | 0.6729 | 165.29 |
|  | 4 | caveola (GO:0005901) | 0.0004167 | 0.1858 | 64.94 |
|  | 5 | centrosome (GO:0005813) | 0.2263 | 1.000 | 3.94 |
|  | 6 | microtubule organizing center (GO:0005815) | 0.2461 | 1.000 | 3.59 |
|  | 7 | integral component of plasma membrane (GO:0005887) | 0.1899 | 1.000 | 2.49 |
| KEGG pathway | 1 | TGF-beta signaling pathway | 3.180e-12 | 9.793e-10 | 121.21 |
|  | 2 | Hippo signaling pathway | 1.066e-10 | 1.642e-8 | 68.18 |
|  | 3 | Cytokine-cytokine receptor interaction | 4.164e-9 | 4.275e-7 | 37.11 |
|  | 4 | Fluid shear stress and atherosclerosis | 0.002531 | 0.1949 | 26.16 |
|  | 5 | Signaling pathways regulating pluripotency of stem cells | 0.002531 | 0.1559 | 26.16 |
|  | 6 | Ovarian steroidogenesis | 0.02663 | 1.000 | 37.11 |
|  | 7 | Axon guidance | 0.004246 | 0.2179 | 20.09 |
|  | 8 | Basal cell carcinoma | 0.03412 | 1.000 | 28.86 |
|  | 9 | Human T-cell leukemia virus 1 infection | 0.1141 | 1.000 | 8.30 |
|  | 10 | MicroRNAs in cancer | 0.1527 | 1.000 | 6.08 |
